# Ocrelizumab-Induced Brain Volume Dynamics in Relapsing-Remitting Multiple Sclerosis

**DOI:** 10.3390/ph19060827

**Published:** 2026-05-25

**Authors:** Roberto De Masi, Stefania Orlando

**Affiliations:** 1 Laboratory of Neuroproteomics, Multiple Sclerosis Centre, “F. Ferrari” Hospital, 73042 Casarano, Lecce, Italy; 2 Complex Operative Unit of Neurology, “F. Ferrari” Hospital, 73042 Casarano, Lecce, Italy

**Keywords:** multiple sclerosis, ocrelizumab, brain volume changes, neurodegeneration, neuroimaging, MRI, VBM

## Abstract

**Background and Objectives**: Ocrelizumab significantly reduces inflammatory activity in relapsing-remitting multiple sclerosis (RRMS), but treatment-induced brain volume change and the specific contributions of white matter (WM), gray matter (GM), and cerebrospinal fluid (CSF) compartments to global atrophy and pseudoatrophy remain unclear. We aim to characterize the longitudinal and infusion-related dynamics of brain compartments in ocrelizumab-treated RRMS patients and identify the clinical and time-dependent predictors of these changes. **Methods**: Fifty-one RRMS patients were enrolled in a four-year prospective study. Brain volumes, including WM, GM, peripheral GM (pGRAY), CSF, ventricular CSF (vCSF), total brain volume (TBV) and their respective fractions (WMF, GMF, BPF) were evaluated by absolute time points (baseline to 4 years) and infusion-based intervals (baseline to 8th infusion), before and after each ocrelizumab cycle. Correlations between brain volume measures and the Expanded Disability Status Scale (EDSS), as well as time-dependent variables such as age, age at onset and disease duration (DD) were examined. **Results**: Mean age was 41.62 ± 9.76 years, mean age at onset 28.17 ± 7.85, mean DD 12.89 ± 8.55 years and mean EDSS 3.26 ± 1.5, indicating moderate disability. During the study, vCSF and CSF significantly increased, whereas WM, WMF, TBV and BPF significantly decreased. Notably, these measures exhibited marked, non-linear, and transient changes during the first year of ocrelizumab treatment, consistent with pseudoatrophy, likely reflecting early resolution of inflammation rather than irreversible tissue loss. GM and pGRAY remained relatively stable, with minor early increases. Correlation analyses revealed that higher EDSS scores, older age, later age at onset and longer DD were associated with lower GM, pGRAY and TBV, emphasizing the interplay between disease progression and development of brain volumetric patterns. **Conclusions**: Ocrelizumab-induced pseudoatrophy is predominantly WM-driven and age-dependent, with WM shrinkage being the main contributor to parenchymal loss and secondary widening of CSF spaces. Age remains a powerful predictor of brain atrophy and neurological impairment. These findings provide mechanistic insights into the biological basis of this modulation in RRMS.

## 1. Introduction

Multiple sclerosis (MS) is a chronic autoimmune disease characterized by inflammation, demyelination and neurodegeneration within the central nervous system (CNS). It affects approximately 2.9 million individuals worldwide [1], leading to significant physical and cognitive disability [2]. The pathogenesis of MS involves a complex interplay of immune dysregulation, genetic susceptibility and environmental factors, ultimately resulting in myelin destruction and axonal injury [3]. Over the past few decades, substantial progress has been made in the development of disease-modifying therapies (DMTs) for MS. These treatments aim to reduce the frequency and severity of relapses, delay disability progression and improve patients’ quality of life.

Ocrelizumab (Ocrevus, Genentech, Inc.—Roche Group, South San Francisco, CA, USA), a humanized monoclonal antibody targeting CD20-positive B cells, has emerged as a highly effective therapeutic option for MS. It has demonstrated significant efficacy in reducing disease activity and slowing disability progression in both relapsing forms of MS (RMS) and primary progressive MS (PPMS) [4,5]. Although the immunomodulatory effects of ocrelizumab are well established, its impact on neurodegenerative processes and brain volume changes in MS remains an active area of research. The loss of brain volume, particularly gray matter atrophy, represents a key pathological hallmark of MS and it is strongly correlated with clinical disability [6]. This progressive brain atrophy is thought to result from a combination of neuroinflammation, axonal loss and neuronal degeneration [7]. Previous studies have suggested that ocrelizumab may exert neuroprotective effects beyond its immune-modulatory properties. Several clinical trials have reported a significant reduction in the rate of brain volume loss, such as whole brain, white and cortical gray matter, thalamic, and cerebellar volume loss, in patients treated with ocrelizumab compared with those receiving placebo or other DMTs [4,5,8,9,10]. These findings raise important questions regarding the potential mechanisms underlying ocrelizumab-induced brain volume changes. Understanding the dynamics of ocrelizumab-related brain volume changes is crucial for elucidating its therapeutic benefits and optimizing treatment strategies in MS. Investigating the relationships among ocrelizumab treatment, brain volume changes and clinical outcomes may provide valuable insights into the underlying mechanisms of neuroprotection in MS. Moreover, such information could facilitate the identification of potential biomarkers for treatment response and contribute to the development of personalized therapeutic approaches, as indicated in earlier studies [11,12].

In this work, we aim to study the contributors and predictors of ocrelizumab-induced brain volume changes in patients with MS, using advanced neuroimaging techniques, including MRI and voxel-based morphometry (VBM). To this end, by analyzing longitudinal MRI data from a well-characterized cohort of MS patients receiving ocrelizumab, we quantified and characterized changes in regional brain volume over time. Furthermore, we aim to investigate the correlations between clinical and time-dependent variables with the main brain volume measures. Recently, our group and the Weideman group have dealt with the topic of how time-dependent variables influence drug response, as well as disability progression, pointing to age as the main contributor to DMT response in MS [13,14,15].

The findings of this study have the potential to deepen our understanding of the neuroprotective effects of ocrelizumab in MS and contribute to the growing body of knowledge regarding its therapeutic mechanisms of action. Furthermore, our results may improve comprehension of the pathophysiological processes underlying brain volume loss in MS and support the development of tailored and patient-specific treatment strategies.

## 2. Results

We enrolled 51 patients, of whom 32 were female and 19 were male, affected by RRMS. The female-to-male ratio was 1.68:1.00. Mean age was 41.62 ± 9.76 years (95% CI: 38.37–44.88), mean age at onset was 28.17 ± 7.85 years (95% CI: 25.47–30.87), mean disease duration (DD) was 12.89 ± 8.55 years (95% CI: 9.95–15.82), mean Expanded Disability Status Scale (EDSS) was 3.26 ± 1.5 (95% CI: 2.74–3.77). These demographic and clinical characteristics of the study population are summarized in Table 1.

**Figure 1:**
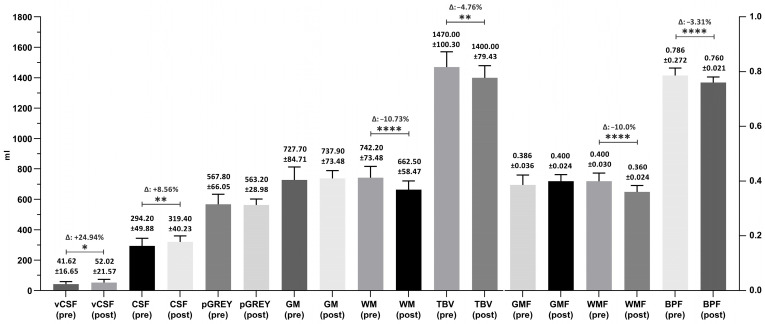
Pre- and post-ocrelizumab brain volume changes. Mean brain region volumes (on the left) and relative fractions (on the right) calculated before ocrelizumab treatment initiation and after the four-year follow-up period. All volumes are expressed in ml as a mean ± SD with 95% confidence intervals. Note the statistically significant difference of vCSF (*p* = 0.0181), CSF (*p* = 0.0047), WM (*p* < 0.001), TBV (*p* = 0.001), WMF (*p* < 0.001), BPF (*p* < 0.001), and the pre-/post-ocrelizumab positive (increase) or negative (decrease) difference expressed as +Δ% or −Δ%, respectively. vCSF: ventricular cerebrospinal fluid; CSF: cerebrospinal fluid; pGRAY: peripheral gray matter; GM: gray matter; WM: white matter; TBV: total brain volume; GMF: gray matter fraction; WMF: white matter fraction; BPF: brain parenchymal fraction. * = *p* < 0.05, ** = *p* < 0.01, **** = *p* < 0.0001.

Mean values of ventricular cerebrospinal fluid (vCSF), cerebrospinal fluid (CSF), peripheral gray matter (pGRAY), gray matter (GM), white matter (WM), total brain volume (TBV) and gray matter fraction (GMF), white matter fraction (WMF), and BPF were calculated before ocrelizumab treatment initiation and after the four-year follow-up period. Statistical differences in vCSF and CSF, WM and WMF, TBV, and brain parenchymal fraction (BPF) were significant between baseline and the study end (*p* = 0.0181, *p* = 0.0047, *p* < 0.001, *p* < 0.001, *p* = 0.001, *p* < 0.001, respectively). Specifically, vCSF and CSF increased, whereas WM, WMF, TBV, and BPF showed a significant decrease over time. Conversely, pGRAY, GM, and GMF remained stable throughout the study period (*p* = 0.387, *p* = 0.087, *p* = 0.276, respectively). These findings are shown in Figure 1.

**Figure 2:**
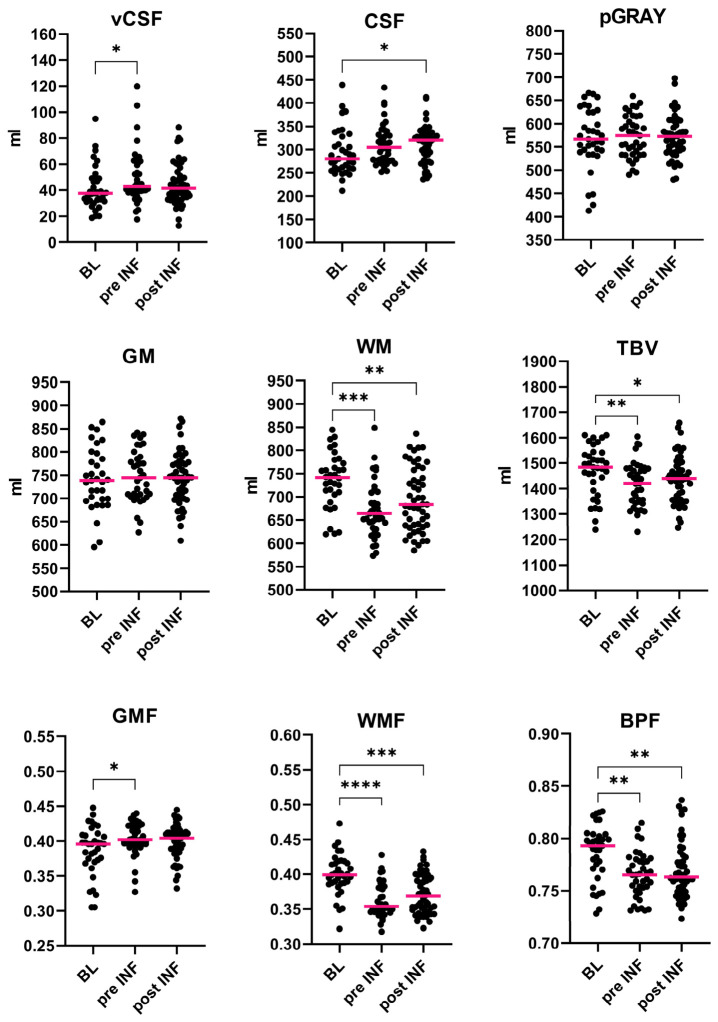
Pre- and post- infusion means of ocrelizumab brain volumes. Mean values were calculated across all infusion cycles, before (pre-infusion) and after (post-infusion) each ocrelizumab administration. Significant differences were observed between baseline and pre-infusion means for vCSF, GMF, WM, WMF, TBV, and BPF, and between baseline and post-infusion means for CSF, WM, WMF, TBV, and BPF (see *p*-values in main text). The pink line indicates the mean value. vCSF: ventricular cerebrospinal fluid; CSF: cerebrospinal fluid; pGRAY: peripheral gray matter; GM: gray matter; WM: white matter; TBV: total brain volume; GMF: gray matter fraction; WMF: white matter fraction; BPF: brain parenchymal fraction. * = *p* < 0.05, ** = *p* < 0.01, *** = *p* < 0.001, **** = *p* < 0.0001.

From the comparative analysis of mean pre- and post-infusion values, we also found significant differences: specifically, in vCSF and CSF between baseline and pre- and post-infusion mean (*p* = 0.0239, *p* = 0.0230, respectively), in GMF between baseline and pre-infusion mean (*p* = 0.0305), in WM and WMF between baseline and both pre- and post- infusion mean (*p* < 0.0001, *p* = 0.0077; *p* < 0.0001, *p* = 0.0001, respectively), as well as in TBV and BPF between baseline and both pre- and post-infusion mean (*p* = 0.0088, *p* = 0.0449; *p* = 0.0012, *p* = 0.0047, respectively). These data are illustrated in Figure 2.

In all cases, we also plotted time-related curves for each observed tissue. Specifically, we evaluated longitudinal brain volume changes at baseline, during the first year, and at 1 year, 2 years, 3 years, and 4 years after ocrelizumab treatment. Furthermore, changes were analyzed relative to the infusion schedule, with MRI scans grouped between consecutive infusions: baseline, between the 1st–2nd, 2nd–3rd, 3rd–4th, 4th–5th, 5th–6th, 6th–7th, and 7th–8th infusions. Analysis of these curves provides pathologically relevant insights into the evolution of brain volume changes over the course of ocrelizumab treatment in MS. In fact, this approach allowed us to evaluate the dynamics of brain volume both over time and relative to ocrelizumab infusion cycles.

**Figure 3:**
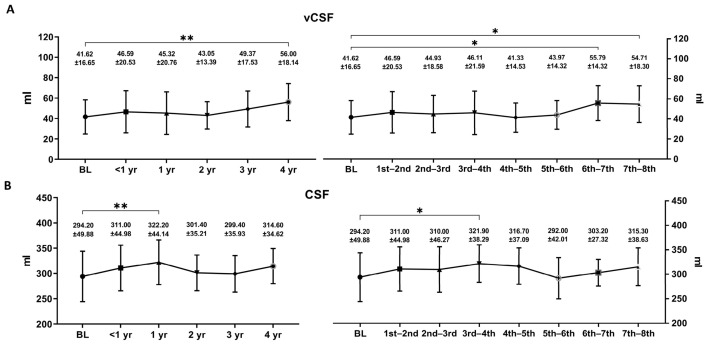
Longitudinal changes in (**A**) vCSF and (**B**) CSF in MS patients treated with ocrelizumab. Time points are shown in absolute follow-up (on the left): BL = baseline, <1 yr = within the first year, 1–4 years = 1, 2, 3 and 4 years after treatment initiation, and infusion-based intervals (on the right): BL = baseline, 1st–2nd = between first and second infusions, 2nd–3rd = between second and third infusions, and so up to the 8th infusion. Note that both analyses revealed similar dynamic patterns, with early increases during the first infusion cycles, modest decreases in intermediate cycles, and further rises in later cycles. Kruskal–Wallis test *p* values: (**A**) *p* = 0.0099, *p =* 0.0269, *p =* 0.0281 for BL vs. 4 yr, BL vs. 6th–7th and 7th–8th, respectively; (**B**) *p =* 0.0052, *p =* 0.0197 for BL vs. 1 yr, BL 3rd–4th, respectively. vCSF: ventricular cerebrospinal fluid; CSF: cerebrospinal fluid. * = *p* < 0.05, ** = *p* < 0.01.

vCSF and CSF showed a net increase over time. However, this increase was not linear: CSF showed an initial rise, reaching a peak after one year, followed by a decrease and subsequent increases over the four-year follow-up. vCSF increased more gradually, reaching its maximum later in the study period. Analyses based on infusion intervals revealed similar dynamic patterns for both measures, with early increases during the first infusion cycles, modest decreases in intermediate cycles, and further rises in later cycles. Figure 3 illustrates these findings.

**Figure 4:**
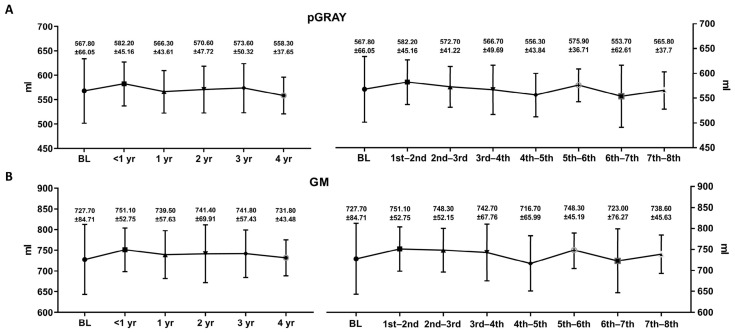
Longitudinal changes in (**A**) pGRAY and (**B**) GM in MS patients treated with ocrelizumab. Time points are shown in absolute follow-up (on the left): BL = baseline, <1 yr = within the first year, 1–4 years = 1, 2, 3 and 4 years after treatment initiation, and infusion-based intervals (on the right): BL = baseline, 1st–2nd = between first and second infusions, 2nd–3rd = between second and third infusions, and so up to the 8th infusion. Note that both analyses revealed similar dynamic patterns, showing a stable trend over time, with a slight overall decrease for pGRAY. Both measures exhibited an initial rise followed by a return to baseline levels, except for a modest peak observed within the first year and between the 5th and 6th infusions. Kruskal–Wallis test *p* values: (**A**,**B**) not significant. pGRAY: peripheral gray matter; GM: gray matter.

pGRAY and GM showed a generally stable trend over time, with a slight overall decrease for pGRAY. Both measures showed an initial rise followed by a return to baseline levels, except for a modest peak observed within the first year and between the 5th and 6th infusions. Figure 4 illustrates these findings.

**Figure 5:**
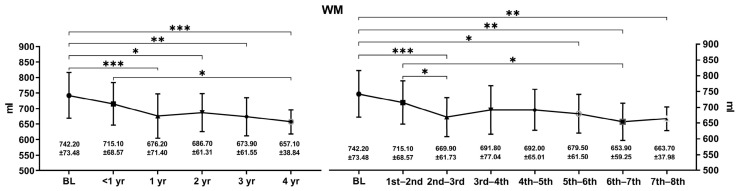
Longitudinal changes in WM in MS patients treated with ocrelizumab. Time points are shown in absolute follow-up (on the left): BL = baseline, <1 yr = within the first year, 1–4 years = 1, 2, 3 and 4 years after treatment initiation, and infusion-based intervals (on the right): BL = baseline, 1st–2nd = between first and second infusions, 2nd–3rd = between second and third infusions, and so up to the 8th infusion. Note that WM showed a consistent reduction over time in both absolute and infusion-based analyses, with a steeper decline observed during the first year of treatment, followed by a more gradual decrease thereafter. Kruskal–Wallis test *p* values: *p* = 0.0007, *p* = 0.0027, respectively for the left and right curves. WM: white matter. * = *p* < 0.05, ** = *p* < 0.01, *** = *p* < 0.001.

WM consistently exhibited a net decrease, with a more marked decline observed during the first year of treatment. Figure 5 illustrates these findings.

**Figure 6:**
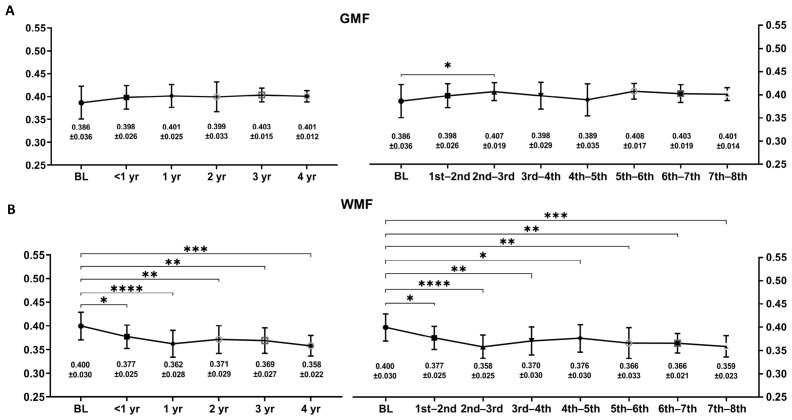
Longitudinal changes in (**A**) GMF and (**B**) WMF in MS patients treated with ocrelizumab. Time points are shown in absolute follow-up (on the left): BL = baseline, <1 yr = within the first year, 1–4 years = 1, 2, 3 and 4 years after treatment initiation, and infusion-based intervals (on the right): BL = baseline, 1st–2nd = between first and second infusions, 2nd–3rd = between second and third infusions, and so up to the 8th infusion. Note that GMF and WMF exhibited opposite trends over time, with GMF showing a slight relative increase—mainly during the first year of treatment—and WMF displaying a corresponding significant decrease, more pronounced in the same period. Kruskal–Wallis test *p* values: (**A**) *p* = 0.0273 for BL vs. 2nd–3rd and (**B**) *p* < 0.001, *p* < 0.001, respectively, for the left and right curves. GMF: gray matter fraction, WMF: white matter fraction. * = *p* < 0.05, ** = *p* < 0.01, *** = *p* < 0.001, **** = *p* < 0.0001.

We also evaluated WMF and GMF of the brain substances analysis in relative terms, expressed as a ratio to the whole brain volume. GMF exhibited a slight relative increase over time, predominantly during the first year of treatment. Conversely, WMF showed a progressive reduction, which was also more pronounced within the first year of ocrelizumab treatment. Figure 6 illustrates these findings.

**Figure 7:**
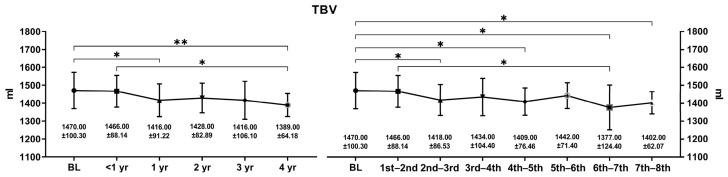
Longitudinal changes in TBV in MS patients treated with ocrelizumab. Time points are shown in absolute follow-up (on the left): BL = baseline, <1 yr = within the first year, 1–4 years = 1, 2, 3 and 4 years after treatment initiation, and infusion-based intervals (on the right): BL = baseline, 1st–2nd = between first and second infusions, 2nd–3rd = between second and third infusions, and so up to the 8th infusion. Note that TBV underwent a net reduction, particularly after the first year from the ocrelizumab start and between the 6th and 7th infusions. Kruskal–Wallis test *p* values: *p* = 0.0181 and *p* = 0.0485, respectively, for the left and right curves. TBV: total brain volume. * = *p* < 0.05, ** = *p* < 0.01.

As regards the global measures of brain volume, TBV showed a marked reduction after the first year of ocrelizumab administration, and this decrease continued over time. Figure 7 illustrates this finding.

**Figure 8:**
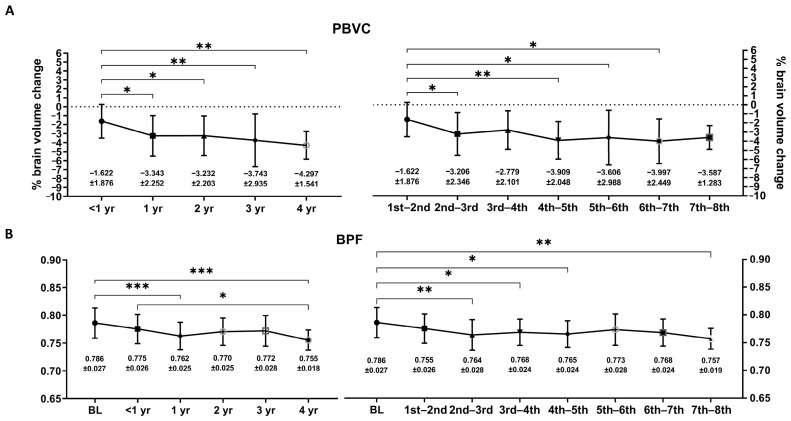
Longitudinal assessment of brain parenchymal loss in MS patients treated with ocrelizumab when (**A**) assessed by SIENA analysis, showing that the majority of tissue loss occurred during the first year of treatment, with an additional reduction over the subsequent three years. (**B**) (BPF) confirmed these findings in relative terms, mainly reflecting tissue loss during the first year of therapy. Time points are shown in absolute follow-up (on the left): BL = baseline, <1 yr = within the first year, 1–4 years = 1, 2, 3 and 4 years after treatment initiation, and infusion-based intervals (on the right): BL = baseline, 1st–2nd = between first and second infusions, 2nd–3rd = between second and third infusions, and so up to the 8th infusion. Kruskal–Wallis test *p* values: (**A**) *p* = 0.0151 and *p* = 0.0397, respectively, for the left and right curves; (**B**) *p* = 0.0028 and *p* = 0.0249, respectively, for the left and right curves. PBVC: percentage brain volume change, BPF: brain parenchymal fraction. * = *p* < 0.05, ** = *p* < 0.01, *** = *p* < 0.001.

We also calculated the percentage loss of brain parenchyma and found that the majority of this phenomenon occurred during the first year of treatment, amounting to approximately 1.6% by the end of this period. SIENA analysis identified an additional 2.7% loss over the subsequent four years. BPF confirmed this parenchymal reduction in relative terms, predominantly occurring during the first year of treatment (see Figure 8).

Additional analyses were conducted to investigate sex-specific differences in brain volumetric changes; however, no statistically significant differences were found between males and females.

**Figure 9:**
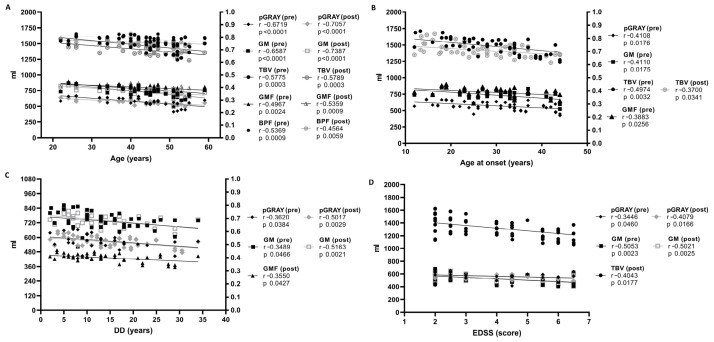
(**A**) Correlations between age and brain volumetric measures in MS patients treated with ocrelizumab. Age showed significant inverse correlations with pGRAY, GM, TBV, GMF, and BPF in both pre- and post-infusion phases. These findings indicate that increasing age is associated with reduced gray matter and total brain volumes, as well as lower parenchymal fractions. (**B**) Correlations between age at onset and brain volumetric measures in MS patients treated with ocrelizumab. Age at onset showed significant inverse correlations with pGRAY, GM, and GMF in the pre-infusion phase, and with TBV in both pre- and post-infusion phases. These findings suggest that a later age at onset is associated with lower gray matter and total brain volumes. (**C**) Correlations between disease duration (DD) and brain volumetric measures in MS patients treated with ocrelizumab. DD showed inverse correlations with pGRAY and GM in both pre- and post-infusion phases, and with GMF in the post-infusion phase, indicating that longer disease duration is associated with reduced gray matter volumes. (**D**) Correlations between EDSS and brain volumetric measures in MS patients treated with ocrelizumab. EDSS showed significant inverse correlations with pGRAY and GM in both pre- and post-infusion phases, and with TBV in the post-infusion phase. Note that the strength of these associations reflects the relationship between higher disability scores and reduced gray matter and total brain volumes. pGRAY: peripheral gray matter; GM: gray matter; GMF: gray matter fraction; TBV: total brain volume; GMF: gray matter fraction; BPF: brain parenchymal fraction.

To further explore the relationships between clinical and volumetric parameters, we investigated the correlations between clinical and time-dependent variables with the main brain volume measures. Specifically, age inversely correlated in both pre- and post-infusion phases, with pGRAY (r = −0.6719, *p* < 0.0001; r = −0.7057, *p* < 0.0001, respectively), GM (r = −0.6587, *p* < 0.0001; r = −0.7387, *p* < 0.0001, respectively), TBV (r = −0.5775, *p* = 0.0003; r = 0.5789, *p* = 0.0003, respectively) and volume fractions of GMF (r = −0.4967, *p* = 0.0024; r = −0.5359, *p* = 0.0009, respectively) and BPF (r = −0.5369, *p* = 0.0009; r = −0.4564, *p* = 0.0059, respectively). Figure 9A shows these results. Similarly, age at onset inversely correlated with pGRAY (r = −0.4108, *p* = 0.0176), GM (r = −0.4110, *p* = 0.0175), GMF (r = −0.3883, *p* = 0.0256) of the pre-infusion phase, and TBV in both the pre- and post-infusion phases (r = −0.4974, *p* = 0.0032; r = −0.3700, *p* = 0.0341, respectively). Figure 9B illustrates these findings. DD showed significant inverse correlations in both pre- and post-infusion phases with pGRAY (r = −0.3620, *p* = 0.0384; r = −0.5017, *p* = 0.0029, respectively), GM (r = −0.3489, *p* = 0.0466; r = −0.5163, *p* = 0.0021, respectively), and with GMF in post-infusion phase (r = −0.3550, *p* = 0.0427). Figure 9C illustrates these correlations. Finally, EDSS showed a significant inverse correlation, in both pre- and post-infusion phases, with pGRAY (r = −0.3446, *p* = 0.046; r = −0.4079, *p* = 0.0166, respectively) and GM (r = −0.5053, *p* = 0.0023; r = −0.5021, *p* = 0.0025, respectively), and post-infusion phase TBV (r = −0.4043, *p* = 0.0177). Figure 9D shows these data.

## 3. Discussion

The mechanisms underlying brain tissue loss in MS remain unclear, likely due to the interplay between inflammatory and neurodegenerative processes. Although inflammatory activity and focal lesion formation contribute to early tissue damage, a substantial component of brain atrophy appears to evolve independently of overt inflammation. DMTs have been shown to exert a clear beneficial effect by reducing relapses and inflammatory lesion burden. However, brain volume changes occurring in patients undergoing treatments are sometimes difficult to interpret, as they may be influenced by pre-existing inflammation at the time of therapy initiation [16,17,18].

The present study aims to investigate the contributors and predictors of brain volume dynamics in MS patients treated with ocrelizumab using two complementary longitudinal approaches: first, according to absolute follow-up time, and second, according to infusion intervals. This dual approach provides, on the one hand, a continuous perspective for evaluating brain changes over time, and, on the other hand, a discrete, point-by-point assessment across each ocrelizumab infusion cycle. To this end, we enrolled 51 RRMS patients, with a female-to-male ratio of 1.68:1, consistent with the general disease population. The mean age of enrolled patients was 41.6 years, the mean age at onset approximately 28 years, and the mean DD was about 13 years. The mean EDSS score was 3.26, indicating a moderate level of disability.

Our results revealed several significant findings regarding changes in brain volume measures and their relationship with clinical and time-dependent variables. First, we observed a statistically significant difference in brain volume measurements between those taken before and after the start of ocrelizumab treatment. Specifically, WM and TBV, along with their fractions, WMF and BPF, showed a significant decrease over time, whereas CSF and vCSF significantly increased. GM and its fractions, pGRAY and GMF, remained relatively stable throughout the observation period. Consistently, the same pattern was observed in the comparative analysis of all pre- and post-infusion mean measurements across treatment cycles. Furthermore, the magnitude of these differences tended to be greater between baseline and pre-infusion means.

CSF and vCSF exhibited a nonlinear increase over time, characterized by an initial rise that peaked after one year of treatment, followed by a transient decrease in intermediate cycles and a subsequent re-increase in later cycles. WM and TBV demonstrated a consistent reduction over time in both absolute and infusion-based analyses, with the steeper decline observed during the initial period of treatment. On the other hand, both pGRAY and GM analyses revealed similar dynamic patterns, showing stable trend over time, with an initial modest increase followed by a return to baseline levels, except for a minor peak observed within the first year. In fact, the dynamics of this substance do not appear to be significantly related to brain atrophy. Consistently, GMF and WMF displayed opposite trends, with GMF showing a slight relative increase, primarily during the first year, and WMF exhibiting a corresponding significant decrease during the same period. These findings clearly indicate a pseudoatrophic effect of ocrelizumab on the brain.

This effect mainly and directly involves WM, with an absolute reduction in its volume and secondary enlargement of the ventricular system, as well as a transient increase in GM volume. This phenomenon likely reflects the resolution of inflammation during the first year of treatment [19], also indicating that WM is the main contributor to brain atrophy. Unlike interferon or natalizumab, which are known to induce pseudoatrophy within the first three months of treatment [20,21], ocrelizumab demonstrates a slower, long-lasting pharmacodynamic action and gradual anti-inflammatory effect.

The transient increase in absolute GM volume observed during early WM volume reduction suggests that pseudoatrophy cannot be solely explained by a reduction of extracellular water content or inflammatory infiltrates. Instead, it may reflect additional mechanisms, such as metabolic recovery or transient cellular expansion following resolution of inflammatory injury. Partial recovery from non-lethal axonal damage within WM tracts may precede retrograde Wallerian degeneration, representing a phase of resilience before irreversible tissue loss. TBV reduction was most marked during the first year of therapy, accounting for approximately 1.6% of parenchymal loss, with a further decline of 2.7% over the subsequent four years, as estimated by SIENA. BPF measurements corroborated this time pattern, confirming that most of the parenchymal loss occurred early after treatment initiation.

Correlation analyses between both clinical and time-dependent variables and brain volumetric measures provided further insights into the structural dynamics of MS and the effects of ocrelizumab therapy. EDSS correlated inversely with pGRAY, GM, and TBV, confirming that higher disability scores are associated with lower gray matter and total brain volumes [22,23]. These correlations are consistent with previous reports, supporting the well-established association between brain atrophy and clinical disability in MS, where axonal loss and cortical atrophy represent key drivers of disease progression.

Similarly, longer DD was found to be associated with lower gray matter volumes and fractions, reflecting cumulative neurodegenerative effects over time. These correlations were largely consistent across both pre- and post-infusion phases, suggesting that the observed relationships primarily capture disease-related structural changes rather than the immediate pharmacological effects of ocrelizumab. Slightly stronger correlations observed post-infusion may indicate a subtle modulatory effect of treatment on brain volumes, potentially mediated by the early resolution of inflammation, consistent with previous reports that DMTs attenuate, but do not completely arrest, brain atrophy in MS [24].

Both of the other time-dependent variables, age and age at onset, correlated inversely with several brain volumetric parameters, including pGRAY, GM, GMF, TBV and BPF, in line with previous reports [25,26]. The magnitude of these correlations is higher for age, thus representing the main predictor of brain atrophy and disability in MS patients treated with ocrelizumab. Specifically, our findings confirm that older age, whether at the time of assessment or at disease onset, is known to exacerbate neurodegenerative processes, contributing to cumulative tissue loss and reduced parenchymal integrity in MS patients, following a well-defined pattern in the dynamics of brain volume changes. This pattern identifies the WM compartment as the main contributor and driving process of brain atrophy. In contrast, the gray-matter compartment remains largely stable and appears to play a minimal role in global atrophy, although it is sensitive to the pseudoatrophic phenomenon observed shortly after DMT initiation. Finally, the fluid compartment gradually enlarges over time, reflecting parenchymal loss and remodeling with adaptation of ventricular size and periencephalic spaces. Notably, the observed patterns were consistent in both long-term longitudinal and infusion-based analyses, indicating that ocrelizumab exerts a sustained anti-inflammatory effect with stable compartment-specific dynamics throughout the duration of treatment.

Overall, these results could have important implications for future research and the clinical management of RRMS patients undergoing ocrelizumab therapy. In fact, a deeper understanding of the dynamic brain volume changes and their associations with clinical variables may help optimize treatment strategies and improve patient outcomes.

The limitation of the study lies in the relatively small sample size, which may limit its generalizability. Nevertheless, the use of rigorous longitudinal and statistical approaches supports the robustness of the findings. Another limitation is the absence of a direct comparison of brain volumetric changes between MS patients and the control group, but this model is not consistent with the aims of the present study.

## 4. Materials and Methods

### 4.1. Study Design

In a longitudinal, monocentric, clinical and paraclinical real-world study, we enrolled 51 consecutive and unselected ocrelizumab-treated RRMS patients, afferent to the Multiple Sclerosis Centre of the Neurological Department at the “F. Ferrari” Hospital in Casarano, Lecce (Italy). Each enrolled subject underwent the drug infusion every six months. According to the user guide, each ocrelizumab infusion consisted of 600 mg administered in 500 mL of normal NaCl solution, except for the first cycle, which was given in two 300 mg infusions in 250 mL NaCl solution, two weeks apart. At the same time, patients underwent brain MRI and were included in a four-year follow-up program. For each patient, baseline MRI scans were acquired before the start of therapy and all of the MRI scans were divided, after the initiation of drug treatment, both by time and by infusion intervals. Specifically, brain volume changes were evaluated longitudinally using two time-point schemes: (1) Absolute time-based intervals: baseline, <1 year, 1, 2, 3, and 4 years after treatment initiation and (2) infusion-based intervals (for a total of 280 cycles): baseline and between the 1st–2nd, 2nd–3rd, 3rd–4th, 4th–5th, 5th–6th, 6th–7th, and 7th–8th infusions. In addition, mean brain volume values were calculated by averaging all pre-infusion and post-infusion measurements across treatment cycles. This approach allowed for the assessment of brain volume dynamics both over time and relative to following ocrelizumab infusion cycles. Finally, clinical and demographic data were collected, including sex, EDSS and time-dependent variables such as age, age at onset and DD.

### 4.2. Study Population

Subjects’ enrollment took place at the MS Centre of Casarano during routine visits, with reference only to inclusion and exclusion criteria.

Inclusion criteria: Relapsing-remitting MS patients previously treated with first-line DMTs, including interferon and glatiramer acetate, and who had failed therapy, were enrolled at the initiation of ocrelizumab treatment.

All MS patients had been previously diagnosed according to the 2017 McDonald criteria [27] and were imaged during an exacerbation-free period of at least three months.

Exclusion criteria: Any metabolic, cardiovascular, or immunological comorbidity (e.g., atheromatosis or prior stroke, diabetes, arteritis, rheumatoid arthritis, or connective tissue disorders), as well as any local or systemic transient inflammatory or septic condition (such as bacterial infections, cold, cough, influenza, exanthematous diseases, viral infections, relevant trauma, smoking, or obesity). Patients receiving any non-MS treatment within at least three months prior to enrollment, as well as those showing abnormal cell counts at the hemocromocytometric examination before study entry, were also excluded.

### 4.3. MRI Protocol

MR imaging of the MS patients was performed on 1.5-T Philips MR apparatus (180 mT/m) (Achieva, Philips Medical Systems, Best, The Netherlands), in accordance with international guidelines [28]. The acquisition sequence types were SE T1–TSE T1 MT–BRAIN VIEW FLAIR 3-D; acquisition time 2.170–3.070–4.140; field of view 230 3183 mm AX–250 3250 FLAIR SAG–180–200 3180 mm COR MT; orientation: TRA–COR–TRA; alignment: TRA–COR–TRA; and voxel size: 0.89/0.88/4–0.56/0.56/4–0.31/0.31/0.6, respectively. Repetition time (TR) was 450–614–4800; echo time (TE) was 15–12–307; and inversion time (TI) was −/−/1660. The flip angle was 69–90°−/, and the NEX was 1–2–2. SENSE parallel imaging method and contrast enhancement (Gadovist single dose, 10 min post administration) were used.

The MRI post-processing consisted of the quantification of brain region volumes and global and selective brain atrophy. Specifically, the brain parenchymal fraction (BPF) was determined according to Rudick’s method [29]. Furthermore, the gray compartment—GM, its fraction (GMF) and pGRAY; the white compartment—WM and its fraction (WMF); the fluid compartment—CSF and vCSF; and TBV were also calculated on T1-weighted images, using the “SIENAX” tool of FSL package software (version number 6.0.0, created by the Analysis Group, FMRIB, Oxford, UK).

Brain volume changes were automatically calculated using the voxel-based morphometry methodology. In particular, total brain volume changes were also calculated with the “SIENA” tool of FSL software for longitudinal observations throughout the evaluation of the percentage brain volume change (PBVC), as appropriate, and the “SIENAX” one for calculating CSF, vCSF, WM, WMF, GM, GMF, pGRAY, TBV, and BPF. MRI images included T1w, T2w, FLAIR, 3DT1, and 3DFLAIR. All measurements were expressed in ml. Axial images (ax) were acquired from all T1- and T2-weighted sequences, axial and three-dimensional images (3DFLAIR) were acquired from FLAIR sequences. Among the image transferring systems, 3DSlicer (version number 5.0.0) and OsiriX (version number 5.8.2) software programs were used as DICOM nodes, to manage the resonance images once acquired and sent from the NMR apparatus. Finally, all scans were acquired using the same scanner and standardized imaging protocol, underwent visual quality control assessment, and automated segmentation outputs were reviewed.

### 4.4. Statistical Analysis

Statistical analysis was performed with the GraphPad Prism9 software. For descriptive statistics, the non-parametric Wilcoxon–Mann–Whitney test was applied to examine the differences between means; for inferential statistics, the non-parametric Spearman test was used to determine the correlation between variables as well as linear regression analysis; the Kruskal–Wallis test was used for multiple comparisons, with Dunn’s test correction applied, where appropriate.

## 5. Conclusions

In conclusion, our results provide new evidence on the global atrophy dynamics during the ocrelizumab treatment in RRMS. The treatment is associated with significant non-linear, longitudinal changes in WM, CSF, and parenchymal fractions, while GM remains substantially stable. Brain atrophy is directly related to disability and age in MS. Pseudoatrophy appears to be predominantly WM-driven, age-dependent, and temporally extended, reflecting the long-lasting anti-inflammatory effect of the drug. A deeper understanding of these dynamics could improve the interpretation of MRI biomarkers and inform therapeutic decision-making in clinical practice.

## Figures and Tables

**Table 1:** Demographic and clinical variables of study MS population. Data are shown as mean ± SD with 95% confidence intervals.

N = 35	Mean ± SD (95% CI)
Female-to-male sex ratio	1.68:1.00
Age (years)	41.62 ± 9.76 (38.37–44.8)
Age at onset (years)	28.17 ± 7.85 (25.47–30.87)
Disease Duration (years)	12.89 ± 8.55 (9.95–15.82)
Expanded Disability Status Scale score	3.26 ± 1.50 (2.74–3.77)

## Data Availability

The original contributions presented in this study are included in the article. Further inquiries can be directed to the corresponding author.

## Abbreviations

BPF	Brain parenchymal fraction
CNS	Central nervous system
CSF	Cerebrospinal fluid
DD	Disease duration
DMTs	Disease-modifying therapies
EDSS	Expanded disability status scale
GM	Gray matter
GMF	Gray matter fraction
MS	Multiple sclerosis
pGRAY	Peripheral gray matter
PPMS	Primary progressive multiple sclerosis
RRMS	Relapsing-remitting multiple sclerosis
TBV	Total brain volume
VBM	Voxel-based morphometry
vCSF	Ventricular cerebrospinal fluid
WM	White matter
WMF	White matter fraction
